# ITK: enabling reproducible research and open science

**DOI:** 10.3389/fninf.2014.00013

**Published:** 2014-02-20

**Authors:** Matthew McCormick, Xiaoxiao Liu, Julien Jomier, Charles Marion, Luis Ibanez

**Affiliations:** ^1^Medical Computing Group, Kitware IncClifton Park, NY, USA; ^2^Medical Computing Group, Kitware IncLyon, France

**Keywords:** reproducibility, ITK, insight toolkit, insight journal, code review, open science

## Abstract

Reproducibility verification is essential to the practice of the scientific method. Researchers report their findings, which are strengthened as other independent groups in the scientific community share similar outcomes. In the many scientific fields where software has become a fundamental tool for capturing and analyzing data, this requirement of reproducibility implies that reliable and comprehensive software platforms and tools should be made available to the scientific community. The tools will empower them and the public to verify, through practice, the reproducibility of observations that are reported in the scientific literature. Medical image analysis is one of the fields in which the use of computational resources, both software and hardware, are an essential platform for performing experimental work. In this arena, the introduction of the Insight Toolkit (ITK) in 1999 has transformed the field and facilitates its progress by accelerating the rate at which algorithmic implementations are developed, tested, disseminated and improved. By building on the efficiency and quality of open source methodologies, ITK has provided the medical image community with an effective platform on which to build a daily workflow that incorporates the true scientific practices of reproducibility verification. This article describes the multiple tools, methodologies, and practices that the ITK community has adopted, refined, and followed during the past decade, in order to become one of the research communities with the most modern reproducibility verification infrastructure. For example, 207 contributors have created over 2400 unit tests that provide over 84% code line test coverage. The Insight Journal, an open publication journal associated with the toolkit, has seen over 360,000 publication downloads. The median normalized closeness centrality, a measure of knowledge flow, resulting from the distributed peer code review system was high, 0.46.

## 1. Introduction

The essential feature of the scientific method is the practice of verification of reproducibility (Popper, 1934, 1963). The large majority of research activity today is focused on generating novelty, and only in exceptional cases, concerned with the verification of reproducibility (Nielsen, 2011; Begley and Ellis, 2012; Nature, 2012a,b,c; Couzin-Frankel, 2013; Vasilevsky et al., 2013). The practice of peer-review has been assumed to be a suitable replacement for the verification of reproducibility, a mistake by which experimental work has been replaced by thought experiments and opinion-based evaluations that do little to further the scientific enterprise (Couzin, 2006; Baker, 2006; Prinz et al., 2011; Russel, 2013; Collins and Tabak, 2014). This drift has denigrated, what used to be scientific work, back into the practice of the “natural philosophy” in which we simply imagine models of the natural word and evaluate them based on aesthetic appeals and desire for self-consistency (Kuhn, 1962).

The National Library of Medicine's Insight Segmentation and Registration Toolkit [Insight Toolkit (ITK)] was conceived in 1999 to support analysis of The Visible Human Project data. In order to maximize its impact, the project embraced an open source development model from its inception. Not only was the project successful in its original objective, it also extended far beyond its original goals and became a foundational component of many National Institutes of Health (NIH) research projects, as well as evolved into a technology underlying many medical image analysis commercial products worldwide.

ITK has made it possible to restore the true practice of the scientific method in the field of medical image analysis. By providing a common platform in which image analysis algorithms and processing techniques can be implemented and can be freely disseminated, ITK empowers all to verify the experimental work of image analysis research activities. This requires that researchers adhere to the true practice of the scientific method and publish the full details of their methodology, including the source code, data, parameters and auxiliary documents that are required for a third party to independently repeat the work and verify its findings (Ince et al., 2012; Collins and Tabak, 2014).

In 2005, The ITK community created a scientific journal, the Insight Journal, to fulfill the practice of the scientific method. In this journal, all articles are required to provide the full set of source code, data and parameters needed to reproduce the finding of the authors. These materials are immediately made available to readers and reviewers, empowering them to perform such verification with minimal effort and minimal loss of information.

Other journals, in particular Frontiers, PLoS, and more recently Nature, have embraced this restoration of the true practice of the scientific method (Nature, 2012c, 2013; Stodden et al., 2013). With the support of the Reproducible Research movement, these progressive publication venues are creating the conditions for a new age of enlightenment in which the methodologies of practical research work will not be subject to secrecy. Nor will they be subject to the veil of suspicion caused by many incidents of scientific fraud and data manufacturing that have been reported in recent months (Couzin, 2006; Begley and Ellis, 2012; Mobley et al., 2013; Sandve et al., 2013).

This article describes the methods employed in ITK to enable reproducible research in the medical imaging community, including version control, peer code review, an online dashboard to display test results, community enablement for sustainability, modularization, and the Insight Journal. Next, the results of these methods are presented, including test code coverage, the impact of peer review on code quality, knowledge propagation implicit in a graph representation of the code reviewers, and contributions and utilization of the Insight Journal. Finally, a high-level reflection on these experiences is presented.

## 2. Materials and methods

The ITK repository currently contains over 2.5 million lines of source code (including 1.2 million lines of third-party code added to the repository). Contributions to this repository can be measured by the number of logical changes made to the code, also known as commits, and the number of source code line additions or deletions. In its 14 years of history, ITK has received 37,626 commits, in 13,184 files, by 209 different authors.

Ohloh.net (Ohloh.net, 2013) is a public directory of open source projects that performs analytics on the code history of communities surrounding projects. According to its Project Cost Calculator, the effort in the toolkit is an estimated 730 person-years, amounting to an estimated cost of 40 million dollars given an average salary of $55,000 per year. As of October 29, 2013, the combined subscribers to the *community*, *insight-users*, and *insight-developers* mailing lists are 2698. The lists average 326 messages per month from October 2012 to October 2013.

### 2.1. Reproducibility assurance practices

The practice of reproducibility verification requires that the software platforms and tools used to support research activities be built in an environment that ensures their high quality, consistency and reliability. This leads to a continuous interplay between correctness and consistency. More explicitly, it would be ideal to expect that running an experiment multiple times would yield the same results consistently. It is ideal if this holds as more recent versions of software tools are used to support the given experiment. However, at the same time, software goes through a continuous process of improvement, modification and correction. Therefore, changes are flowing on a steady manner into the code base. To balance the benefits and challenges of this interplay, it is essential to use both a set of software quality support tools, and a set of community practices that make proper use of such tools.

To ensure the code quality of the toolkit and the growth of the ITK community, adaptation to modern software practices are necessary. In particular, the software quality process relies on the combination of an automatic testing system (CDash), a version control system (Git), a code review system (Gerrit).

#### 2.1.1. Open dashboard: CDash

ITK has a stringent system of quality control that uses a combination of unit testing, regression testing, multi-platform verification, and continuous integration. The collection of unit tests are executed nightly by computers contributed by community members around the world, and reporting to a central online dashboard that summarizes the results. This web-based dashboard system (CDash) (CDash, 2013) ensures ITK's software quality as developers world continuously make changes to the code base. Build status and regression test status are visualized in a tabular form. The dashboard is an important coordination and communication tool that empowers developers to share the results of a local test with other developers by pushing them to the online summary pages.

Continuous builds triggered by patches submitted to the Gerrit code review system also give feedback to developers on the impact of recent changes. Nightly builds of the project spanning a wide variety of platforms and configurations ensure that ITK can be built on a diversity of operating systems and hardware.

#### 2.1.2. Source code version control: Git

Version control is an essential practice that must be applied to all software used to support the quest for knowledge in a reproducible research environment. A good source code version control system allows developers to easily keep track of the entire history of the software development. Researchers can reference the complete state of experimental source code, and a reviewer can reliably and easily regenerate that archived state for examination. Git is an open source, distributed version control system designed to perform with speed and efficiency. Its features include easy local branching, convenient staging areas, and multiple workflows, which are particularly useful for large open source projects like ITK.

The ITK community embraced the used of Git as part of the modernization activities leading to ITKv4 in 2010. When migrating the source code repository from CVS to Git, the history of the development was preserved and a simple Git workflow was customized for ITK developers. The adoption of Git empowered ITK developers to easily create flexible workflows of branching and merging. It also allowed developers to make commits in local repositories, and hence experiment independently with variations on the software.

Despite the initial steep learning curve to learn commonly used Git commands, Git provides a superior, welcoming collaboration platform for the community. It has become the new standard for source code version control. And more importantly, great tools that are base on Git are accessible to ITK, such as the peer code review system: Gerrit Review.

#### 2.1.3. Open review system: Gerrit review

In the context of reproducible science, the detailed description of the methods and materials used to perform an experiment are a fundamental piece of the article that disseminates the results. As research activities become more and more dependent on software for the preparation, execution and analysis of experiments, it becomes necessary to include all the details related to the software as part of a reproducible publication. Given the complexities of software implementation, mere algorithmic descriptions, or even pseudo-code, are not sufficient to ensure reproducibility through reimplementation. Only the delivery of original source code can provide enough assurances that the recipient of the materials will be able to replicate the reported work with a reasonably limited amount of effort.

The code must therefore be subject to peer review processes similar to the ones currently used for scientific publications. Such reviews should examine both the quality and correctness of the code, as well as proceed to verify the reproducibility of the code through its actual execution.

Recently, recognition of software's importance for the progress of science has elicited movements such as the “Science Code Manifesto” (Barnes, 2011). While a number of journals are beginning to adopt practices similar to The Insight Journal, where code is submitted along with the article, evaluation tools and procedures for peer review of the code are not in parity with those used for the article. Commonplace practices have evolved to facilitate article review such as evaluation rubrics, instrumentation of the text with line and page numbers for reference during discussion, and a process to distribute an article to reviewers and communicate author replies. However, the technical nature of code solicits greater technical capabilities of the tools and methods used to evaluate its reproducibility.

While this problem is multi-faceted, some progress has been made through ITK's adoption of the Gerrit Code Review system. Gerrit is an open source project maintained by the Google Android mobile phone project. This system enables a large community of developers to inspect and comment on the code changes that are proposed for inclusion in a software system. Not only has the ITK community embraced the use of Gerrit, it has also contributed fixes and features back to the upstream Gerrit project.

Gerrit is implemented as a combination of a web-based front end with a Git repository backend. Most of the interactions that developers have with Gerrit, happen with the web-based front end server. The Gerrit server provides a mechanism to effectively evaluate code changes, obtain and test those changes locally, and perform notification and transmission of the changes and comments for authors and reviewers, as well as management of the system to accept merges.

Gerrit is a technology that is built around the Git distributed version control system. With Git, contributors can independently develop and test patches that are put in topic branches created out of the ITK master branch. The patch can be shared with the community by pushing the topic branch to the Gerrit server (Gerrit, 2013). Once in the server, the change is publicly accessible via the web-based front end. Developers can then use a web-browser to see side-by-side differences that are easily identified with color-highlighting on a file-based diff page.

From the web interface, contributors can request the attention of specific reviewers to comment on their patch. As a convenience, the web-interface provides auto-completion of names for any community member that has registered an account with the server. The reviewers for a given change can be added or removed throughout the review process by any community members, and they will be notified via email of any new comments or change revisions. In the Gerrit system, every change is identified by a unique Change Id, which allows multiple revisions, also known as Patch Sets, to be uploaded consistently in response to comments.

The discussion of the change between author and reviewers occurs via three mechanisms: overall comments on the change, inline comments, and numerical ratings. Overall comments conveying general remarks can be added per Patch Set, and the history of comments is retained and easily navigated. Questions and suggestions can be directed at specific sections of the code with the inline comments. Whether the code can reproducibly be built and pass tests is indicated by the reviewer with a numerical Verified score, and overall evaluation is indicated with a numerical Code Review score. With the Gerrit Code Review system, reproducibility is improved through continual refinement of the corpus of ITK knowledge, as embodied in the code repository, through experimentation as implemented in the unit tests and through peer review as exercised in the code reviews.

### 2.2. Sustainability

In order to facilitate the long term viability of a project, it is essential to cultivate an active community around it. The adoption of proper cultural practices by the community ensure the long term quality and sustainability of the project, in a manner that is consistent with the principles of reproducibility and therefore, with the practices of open science. In addition to proprietary applications, ITK is the foundation for a number of open source analysis tools; a small sampling includes 3D Slicer (Fedorov et al., 2012), ANTS (Tustison et al., 2009), Elastix (Klein et al., 2010), ITK-SNAP (ITK-SNAP, 2014), MITK (MITK, 2014), OsiriX (OsiriX, 2014), Seg3D (CIBC, 2014), Vaa3D (V3D, 2014), Voreen (Voreen, 2014), and VV (VV, 2014).

#### 2.2.1. Community enablement

The ITK community has cultivated a number of practices intended to ensure that the demographics of contributors are continuously renewed. In this way, the community remains active and vibrant, and can provide the manpower needed to maintain and improve the ITK. In particular, this involves welcoming new members and training them on technical skills, social rules, governance processes, and cultural practices.

On the training front, the ITK community has been hosting a series of webinars to promote ITK in general and the new features available in ITK version 4 (ITKv4) in particular. The webinar videos have been posted publicly, and have received more than 1000 plays so far. As the number of webinars grow, they also start covering more advanced topics, such as the porting of ITK to devices based on the ARM architecture, with webinars such as “Raspberry Pi Likes ITK” and “Raspberry Pi Likes ITK with VTK,” that describe how to use ITK in the highly popular Raspberry Pi board.

To empower new developers and lower the barrier of entry for new users, a web site was put in place to host and distribute a large collection of examples on various ITK classes and filters (http://itk.org/ITKExamples).

In a very focused effort to grow the ITK community, an online space called ITKBarCamp (http://insightsoftwareconsortium.github.io/ITKBarCamp-doc/) was created to train new community members on the software technologies that are essential to ITK. This space provides a combination of training materials, such as source code, documentation and video tutorials, that guide newcomers at their own pace through training activities aimed at honing their software development skills.

One of the very active areas in the ITK Bar Camp is a series of participatory, short YouTube.com videos with associated documentation covering various topics related to ITK, including:

1. Mastery of the command line
2. Basic C++ programming skills
3. Good software practices, including unit testing
4. Recommended tools and workflows for ITK development.

So far 29 short tutorial videos have been created, which have received 2234 views and attracted 30 subscribers. ITK Bar Camp materials are hosted in Github as written documentation with references to video archives. Information on previous and current webinars and hangouts can be found at (http://www.itk.org/ITK/resources/webinars.html).

These educational efforts are crucial to attracting, training, and retaining new community members in the long run, and through that mechanism replenish and sustain the community with active members.

#### 2.2.2. Modularization

Since its inception in 2000, ITK was designed as a collection of about seven core libraries and about ten third party libraries. This monolithic organization of the code led over time to very large sub-libraries, as more classes were added to the toolkit. Once the core code of ITK surpassed half-a-million lines of code, it became evident that a more modular approach was needed in order to support the future continued growth of the toolkit.

Such modularization was implemented in 2010–2011 as part of the larger refactoring effort that culminated to ITKv4. As a result of the modularization, the initial monolithic code base of about 12,000 files was partitioned into more than 100 modules. And, as of Oct of 2013, ITK's main code repository contains 137 modules in total.

**Figure 1:**
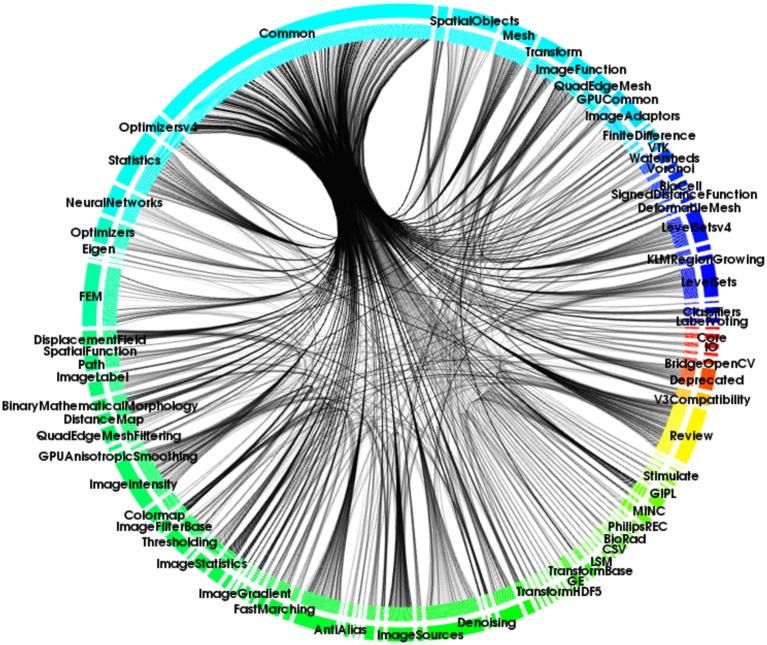
**An illustration of the ITK modules' dependencies**.

Module dependencies (see Figure 1) were identified and explicitly declared in the CMake-based (CMake, 2013) configuration system. By making the CMake-based configuration system aware of the new ITK modularization, ITK adopters became enabled to select, at configuration time, the pieces of ITK that they wanted to use in their own projects.

Support for adding Remote Modules was also built into the ITK modularization infrastructure. These are modules that can be coupled with a particular ITK source code installation, whose source code are distributed separately from the main ITK repository. By making it possible to easily integrate new modules, the Remote Module infrastructure enables fast dissemination of research code through ITK without increasing the size of the main repository. These Remote Modules can be exchanged across members of the community based upon interest and need, which accelerates the rate of dissemination of emerging modules as well as helping those modules mature faster through the rapid use, testing, and commentary by early adopters. Currently there are four ITK remote modules hosted on GitHub.

**Figure 2:**
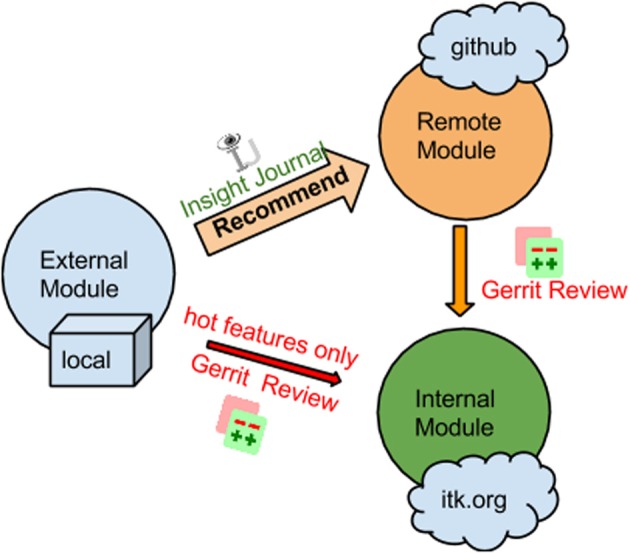
**Code contribution process in ITK**.

The modularization effort significantly improves the extensibility of the toolkit and lowered the barrier of contribution. A recommended contribution process is illustrated in Figure 2. All new modules can be dropped into the ITK source tree as external modules for testing, and they should be submitted to Insight Journal for review. Once the External module passes dashboard testing and peer review, it can be submitted as a Remote module. As a result, it will be disseminated from ITK itself while its source code is maintained independent of the main ITK repository. Specifically, the source code of a Remote module can be downloaded via CMake at configuration time, which makes it a convenient method to distribute modular source code without increasing the size of the main repository. After the Remote Module has experienced sufficient testing and community members express broad interests in the contribution, the submitter can then move the contribution into the ITK repository via Gerrit code review. It is possible but not recommended to directly submit a module to Gerrit for review without submitting to Insight Journal first. An Insight Journal article describing the background, functionalities, and the design behind the module is a great way to provide extra documentation.

### 2.3. Open publication: the insight journal

ITK was conceived as a usable encyclopedia of image analysis algorithms that are of particular utility to the medical imaging community. Given the rapid pace at which technology develops in this area, and the proliferation of both generic and specialized image analysis algorithms, it is important for ITK and its community to continuously update the content of the toolkit by adding new algorithms while simultaneously improving and extending existing ones. To do this, the ITK community relies on contributions made by its members as they use the toolkit to support their own projects and run into situations where additions and improvement are required for them to achieve their goals. In order to absorb these contributions, the ITK community has used the Insight Journal (ITK, 2013) since 2005. The online Insight Journal publishes practical articles written by developers for developers and requires those articles to be fully reproducible.

The Insight Journal is a free, open-access publication covering the domain of medical image processing and visualization. It enables community members to publish their contributions to medical projects for open peer-review, full reproducibility verification, and open-rating by readers worldwide.

The Insight Journal is currently the only technical publication in the domain of image analysis that not only allows but also requires verification of reproducibility as part of the submission and review process. The importance of restoring the practice of reproducibility verification in scientific research has resurged in recent years in light of worrisome findings of inconsistency, lack of quality and even fraud in what were otherwise considered to be high-quality publications (Begley and Ellis, 2012). By adhering to a reproducibility verification requirement, the Insight Journal ensures that community members get rapid access to reliable publications that include open source software that they can readily use in their projects.

The Insight Journal now accepts ITK module submissions. This feature empowers community members to take advantage of the new modular structure in ITK and makes future code integration easier. Readers can then download Insight Journal articles and directly plug them in as Remote Modules on their local ITK installation.

## 3. Results

### 3.1. Contribution statistics

**Figure 3:**
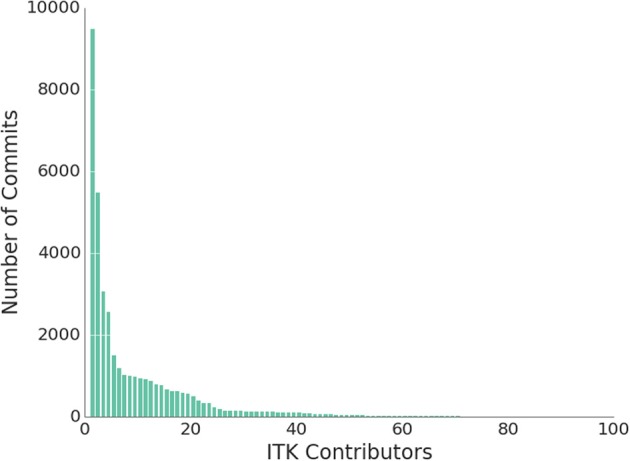
**Histogram of ITK contributors by number of commits in the repository since 2000**.

Figure 3 shows the distribution of contributions to the source code of ITK. Every bar corresponds to an individual contributor. The height of the bar represents the number of commits that this contributor has authored. The figure shows the top 100 contributors, out of a total of 207. The ITK community follows a typical power-law distribution, where a concentrated number of contributors are responsible for the majority of the commits, while a large number of contributors make small contributions. This phenomenon is known as the “Long Tail,” and it is quite important for the structure of the community. The long tail of contributors who make small changes are an important strength of the community since they tend to provide the majority of the quality control after versions of the software have been released. The head and the tail of the distribution have interacting dynamics that complement each other and ensure that innovation makes its way into the software, while at the same time it matures and gets adjusted to the true needs of the larger number of adopters.

### 3.2. Software quality assurance

When integrating the contributions of a large number of community members, it is vital to have in place a quality control mechanism. This eliminate defects in the code before they are introduced into the repository, and it also ensures consistency and coherency across the toolkit. The quality assurance infrastructure of ITK is implemented by a combination of several interacting tools. In particular: the configuration system CMake, the online quality control dashboard CDash, the revision control system Git, and the code review system Gerrit. This infrastructure is complemented by coordination and communication tools such as mailing lists, wikis, weekly phone calls, and regular online videoconference meetings.

The life cycle of a code change starts in the communication channels when community members raise issues in the code. These issues might relate to lack of correctness, lack of desirable features, run-time performance bottlenecks, lack of support for specific platforms, or problems with specific types of data. Typically, the issues are discussed among community members until it is determined that changes in the code are required, and that one or several community members will take on the task of implementing the required changes.

Once community members prepare initial versions of the required changes, they commit them in their local clones of the Git repository, from where they can submit them to the peer code review tool, Gerrit. A group of two or three reviewers are invited to review and test the code contributions and a conversation ensues, where the submitters and reviewers iron out any limitations or imperfections in the suggested changes. During this exchanges, test builds are generated by an automated, cross-platform testing system, which are submitted to the CDash dashboard where they are publicly available for inspection.

When the reviewers and submitters reach an agreement on the final version of the changes, they are merged into the official repository and are incorporated into the code base for the upcoming release.

This process is supported by stringent testing requirements. In ITK, it is expected that every C++ class should have its own unit test and that algorithmic combinations should have additional tests. As a consequence, ITK carries about 2400 unit tests, which result in a code coverage higher than 84%.

### 3.3. Open science publication and review

**Figure 4:**
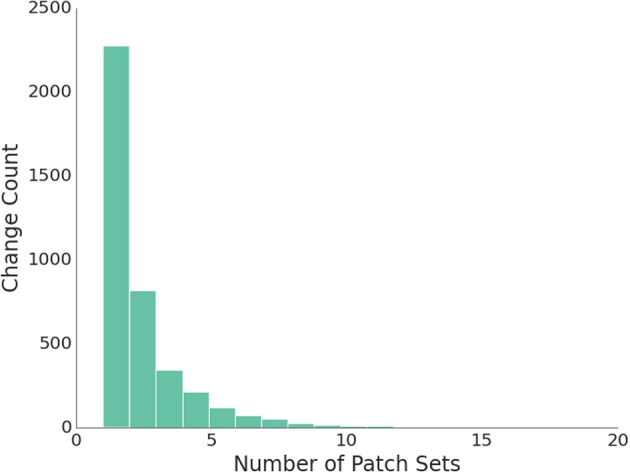
**Histogram of the number of revisions (Patch Sets) for a given change**.

In the 3 years that the community has applied the Gerrit code review system, 4005 changes have been submitted to the Gerrit review server and 6122 reviews were performed. As a matter of policy, all merged changes should have at least one review, but the number of iterations on a change varies flexibly. This results in a roughly negative exponential distribution in revisions, as evident in the histogram of revisions in Figure 4. The highest number of reviews for a single change was 38.

Two direct but notable conclusions follow from this data. First, at least one other person examined and reproduced a proposed change. This certainly exceeds the publication systems where code is never disseminated. And, it likely exceeds validation systems where the code is published, but there are not incentives or checks that reviewers looked at or applied the code. Secondly, the number of Patch Sets greater than one also indicates that improvements were made and errors were identified during this process; coding errors are common even in the context of a scientific software project with experienced developers and quality control systems that far exceed those applied by the typical research scientist.

**Figure 5:**
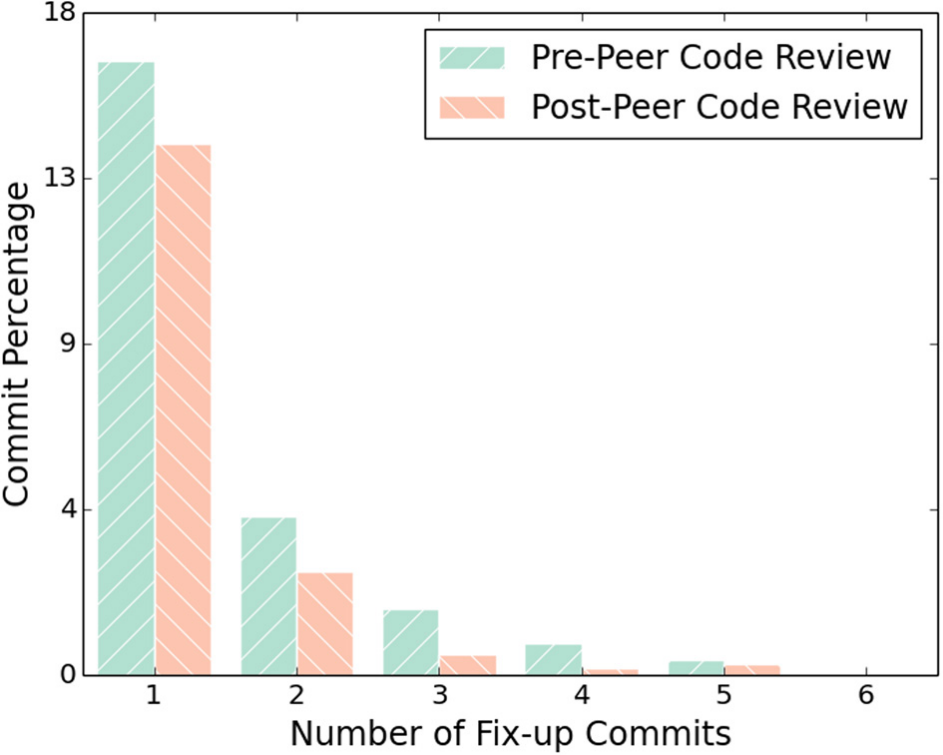
**Fix-up commit percentage before and after peer code review**.

This hypothesis is further supported by Figure 5, in which the number of “fix-up” changes are quantified. A fix-up change is defined in an objective way that roughly captures changes intended to fix bugs introduced in the previous Patch Set. Sections of a patch, traditionally called hunks, which are additions or modifications, are identified, and all commits in the following 5 days are examined. If content in the committed hunk is modified in that period, it is labeled as a fix-up commit. If addition or modification hunks in the fix-up commit are again modified in the following 5 days, then the original change is said to have two fix-up commits, and so on.

We computed fix-up commits in the ITK repository for a 3 year period preceding the adoption of the peer code review system from August 25th, 2007 to August 25th, 2010 to the 3 year period following from August 25th, 2010 to August 25th, 2013. There were 7242 changes during the pre-peer code review period and 4431 during the post-peer code review period. As shown in Figure 5, this reduces the number of single fix-up commits from 16.7% to 14.4% and dramatically reduces the percent fix-up commits by approximately half for higher numbers of fix-up commits.

**Figure 6:**
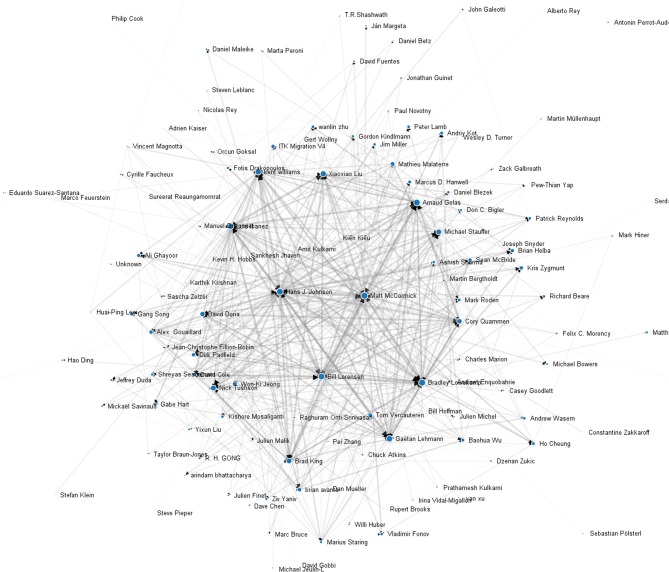
**Peer code reviews graph.** Nodes are individual community members and size of the circle at the node is related logarithmically to the number of changes created by that contributor. The widths of the edges in this directed graph are logarithmically proportional to the number of reviews performed.

The underlying cause of this apparent reduction in errors is suggested by the graph visualization of Figure 6. This graph represents the 3 years of reviews performed by the ITK community. Each node is a community member, and the size of the node is logarithmically related to the accumulated number of changes created by that community member. The edges in the directed graph represent accumulated reviews given by one community to another.

**Figure 7:**
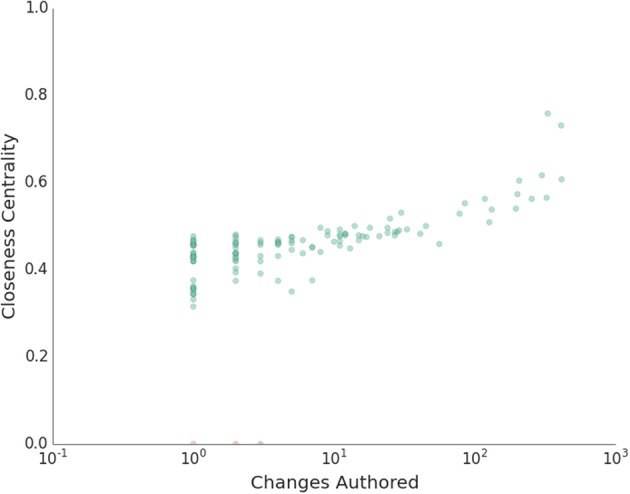
**Normalized closeness centrality of peer code review graph versus the number of created changes.** Different connected components are shown in different colors.

A number of high level observations can be made from this graph. There is a spectrum of node sizes and connectivities that correspond to varying degrees of collaborations. While there are some node pairs that have mutually strong connections, the reviews are generally well distributed. Cohesiveness of the community is evident by the well-connected property of the graph and the closeness of all nodes. This is further quantified in Figure 7. Here an undirected version of the graph is used to compute the closeness centrality of each node against the logarithm of changes created. Closeness centrality is defined as, Freeman (1979)

(1) $$C(u)=\frac{n-1}{\sum_{v=1}^{n-1}d(v,u)},$$

where *d*(*v*, *u*) the shortest path distance from node *u* to node *v*, and it is normalized by *n* − 1 nodes in the graph. This quantifies the reciprocal of the average distance between nodes; i.e., how close the nodes are from each other or how long it will take to spread information from one node to all other nodes sequentially (Newman, 2005). Overall, this measure of communication is rather high across the board, and individuals with a higher number of changes also have a higher centrality measure. There are three outliers, but all other contributors are members of the primary connected component. The median closeness centrality of the primary connected component is 0.46.

The Insight Journal is very active, and since its inception in 2005, it has published 256 articles with 477 public reviews and has more than 2400 subscribed readers. Just the top five most popular articles have been downloaded more than 5000 times each, for a combined number of 43,384 downloads and combined number of 71,500 views. Since August, 2013, there have been over 360,000 downloads of the articles and over 1,790,000 views.

**Figure 8:**
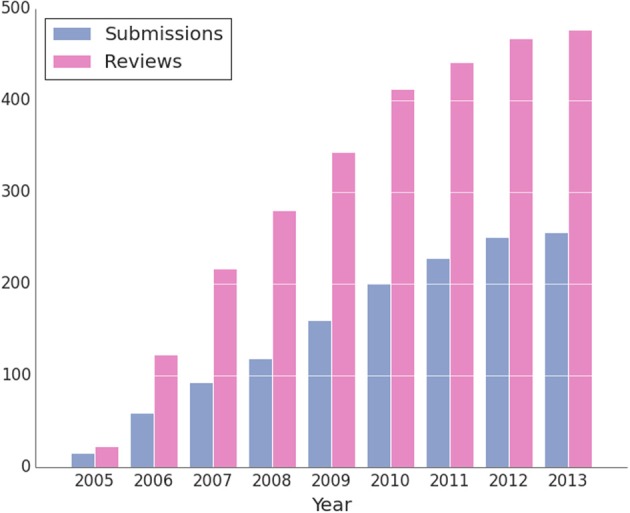
**Insight Journal submissions and reviews over the journal's lifespan since 2005**.

Figure 8 displays the progression of submissions and reviews of articles in the Insight Journal since its inception in 2005. There has generally been a linear increase in submissions and reviews since 2005 with the rate of submissions tapering off since 2010. The number of reviews is approximately double than the number of submissions.

### 3.4. Illustrative analysis

**Figure 9:**
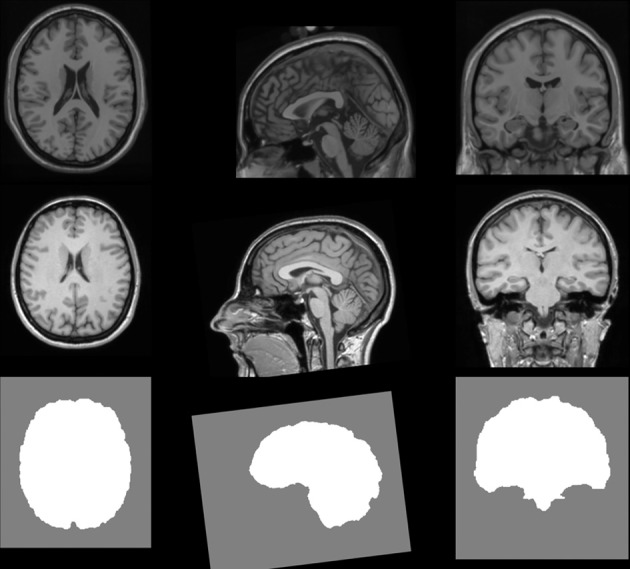
**Input datasets for the skull stripping segmentation task.** Axial, sagittal, and coronal views of the input patient dataset (top row), the reference atlas (middle row), and the segmentation of the reference atlas (bottom row).

In this section, an analysis pipeline is presented to highlight some of the capabilities of the toolkit. Segmentation of the brain from a magnetic resonance image (MRI) of the head is presented on open data and the source code included with the publication. An extension of the skull-stripping algorithm published by Bauer et al. (2011b); Bauer et al. (2011a) available in the toolkit is applied to the Colin 27 stereotaxic 1998 brain model from the McConnell Brain Imaging Center (Holmes et al., 1998) (Figure 9).

The format and type of 3D neural data for this analysis highlight the medical imaging-specific capabilities of ITK. Unlike photographic images that are uniformly 2D with isotropic pixel spacing, medical images are often two, three or more dimensions, have a variety of complex pixel types, like diffusion tensors, an offset from their origin, anisotropic spacing, and an off-axis orientation. To address these challenges, computations in ITK are performed in physical space, which accounts for anisotropic spacing, etc., and are written in such a way that they apply to N-dimensional datasets. Arbitrary pixel types are supported, and a number of standard and medical imaging specific file formats for 2D, 3D, and ND datasets are supported, including BioRad, BMP, CSV, DICOM via the GDCM or DCMTK libraries, Analyze, HDF5, JPEG, LSM, MetaIO, MGH, MINC2, MRC, MGH, NIFTI, NRRD, PhilipsREC, PNG, Stimulate, TIFF, SCIFIO, and VTK.

The skull-stripping solution is initialized by registration of the target patient data set against an atlas that has a brain segmentation. In this case, the publically available XIX atlas (Hammers et al., 2003; Gousias et al., 2008; Bauer et al., 2011a) is applied. A rigid registration followed by an affine registration is performed with a mutual information metric. In addition to integrated, specialized registration algorithm implementations, ITK has a modular registration framework where a choice of transform, optimization strategy, and metric can be paired from a variety of options. The image resampling process, which can be used independently from the registration framework, has a number of interpolation functions available such as nearest-neighbor, linear, b-spline, Gaussian, and windowed-sinc.

Following registration, the transformed atlas mask is used for initialization of the brain segmentation, which is refined with a level set segmentation. ITK contains full-featured level set segmentation implementations along with other segmentation algorithms and tools such as region growing algorithms and classes to evaluate the Dice and Jaccard overlap measures.

**Figure 10:**
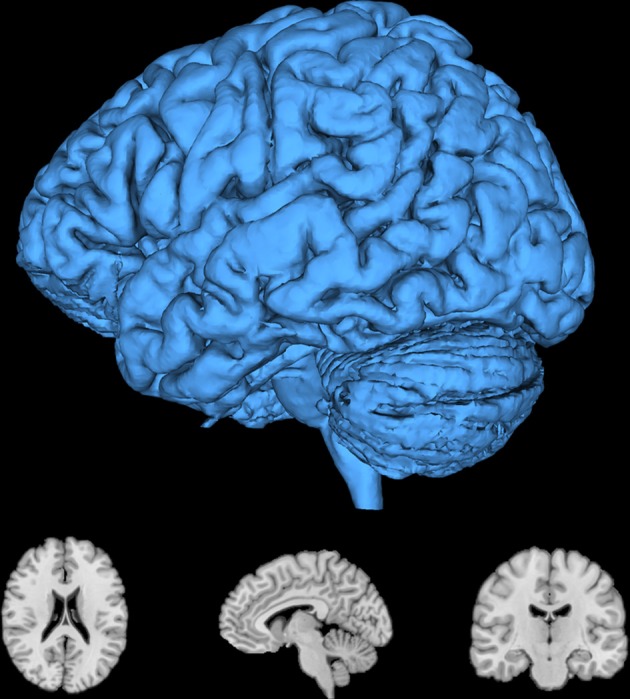
**Result of the brain segmentation of the head MRI from Figure 9.** A surface rendering is displayed along with axial, sagittal, and coronal slices. These images were rendered with 3D Slicer (Fedorov et al., 2012).

The resulting segmentation is refined with mathematical morphology filtering algorithms. Segmentation and registration algorithms in ITK are complimented by a number of advanced edge detection, smoothing, convolution, deconvolution, mathematical morphology, bias correction, distance map, thresholding, and other filtering algorithms. These filters can be chained into an analysis pipeline that will automatically re-execute all the effected filters when an input or parameter changes. The output of the segmentation is rendered in Figure 10.

## 4. Discussion

The code review system helps to eliminate bugs, results in design improvements, assists in the training of new developers, and provides a great communication platform for collaborative development. Since it has so many software quality advantages, code reviews are as critical to ITK as the creation of new features.

The review graph (Figure 6) indicates there is not a binary distribution of “users” and “developers” but there is a continuous transition in contribution and experience by community members. There are not a few nodes from which radiate all knowledge and advancement, but a bi-directional network where knowledge and experience can flow thoroughly. Indeed, all the large nodes have many incoming edges. The graph is also not grouped into islands of isolated knowledge, but it is fully connected with few hops from any given community member to another community member.

These properties are the result of policies and practices that encourage their emergence. The adoption of the Gerrit Code Review tool places an explicit emphasis on code review. Registration for an account is publicly available, and the default permissions allow community members to not only submit changes but also perform reviews. All community members, including the novice ones, are also highly encouraged to perform reviews in project documentation, which promotes the culture of open code review. Second, the policy of requiring a positive code review, even for the most experienced community members, encourages continuous improvement of even senior members while promoting civility. It discourages obstructionism and encourages collaboration by promoting reciprocal constructive criticism.

While the Gerrit Code Review system provides fine-grained and thorough review of code as it enters into the Toolkit, the Insight Journal plays an important role in the high-level introduction and presentation of new algorithms that are often relevant to the Toolkit. While the medical imaging and broader scientific community have recently embraced the open science movement that champions reproducible research through online open access to articles, open source code, and open data, the Insight Journal has had these qualities for many years, and there are lessons to be learned from its experiences.

As indicated by the high number of views and downloads, the utility and value of articles published in the Insight Journal is high. Unlike traditional journal articles that are accessible only to those privileged with a subscription to penetrate their paywall, articles in are available to any researcher with an internet connection. Once the article, code, and data are obtained, they offer much more value to a researcher. Instead of only gathering a small level of information from a suspiciously reported text, the researcher can verify the results, understand the “devilish details” elucidated by the source code, and use both the code and data as a starting point for new avenues of research. Additionally, the researcher may not be interested in the theory underlying the reported algorithm at all—the implementation provides a concrete solution to address a tangential problem at hand. Indeed, algorithms and solutions published in many academic journals are heralded for their ability to address important problems, but solutions often never see translation into pragmatic application because they are irreproducible and false or because they lack an easy to apply manifestation in quality, clean code.

The age of the journal has revealed a practical challenge in source code submitted with articles. Since software is a constantly evolving creature that must be nurtured and maintained, older articles will more often than not fail to build or run, even if they passed their initial quality assurance testing. This can not be avoided as computer architectures progress, operating systems upgrade, and libraries mature. A recent flourishing of technologies such as operating system virtual machines and easy-to-apply container systems may help archive submissions so they can be tested in their originally submitted environment.

While the journal has seen a fair number of submissions, the submission rate has dropped significantly since 2010. In 2010, the funded efforts to support the toolkit were focused on the development of ITK version 4, with decreased attention paid to the broader community. Since many funding agencies have been slow to acknowledge the Insight Journal as one of the traditional journals that drives the academic “publish or perish” career economy, the primary incentive to submit to the journal is impact on a vibrant research community. Consequently, visible activity on the mailing lists and the adoption of published articles into the toolkit drove submission rates prior to 2010. Therefore, to drive activity upwards, efforts must be put forth to increase community vibrancy, give credence to the value of publications in the Insight Journal relative to traditional peer-reviewed journals, and promote and embrace more just alternative incentive mechanisms for funding and career advancement.

It is also notable that the number of reviews is low relative to the number of submissions. Again, incentive mechanisms should improve to reward reviews. It is notable that the journal has also had a non-blinded, open review policy where the names of reviewers are made public. While this increases accountability and transparency, it is known to discourage review activity (van Rooyen et al., 1999; Walsh et al., 2000). While many outstanding articles have been submitted to the toolkit, relatively few have been merged into the toolkit. This reflects the amount of effort required to reproduce another's work during review. To address this significant challenge, two approaches have been taken. First, the standard, pluggable modular system (section 2.2.2), greatly lowers integration barriers. Second, authors are self-enabled, with technologies such as Gerrit, to directly respond to reproducibility barriers discovered during review. However, there is no complete, transient solution to this problem; the scientific community must recognize that reproducible science requires time, effort, and resources.

While an open source software image analysis library like ITK is necessary for open science, it is insufficient to truly enable reproducibility. Data and documentation are also required (Carp, 2012; Vines et al., 2013; Glasziou et al., 2014). Acquisition and location of data is difficult even with the sparse publically available datasets, but accessible indexes such as the Datastore Module in 3D Slicer (Fedorov et al., 2012) are helping to address this issue. The open source nature of ITK, the open access nature of the Insight Journal, and the public sharing of open data in the ITK community, are the three pillars of Open Science that are transforming the way scientific research is done today.

The technological challenges of reproducibility have all been solved. We now require a cultural change by which we must make it unacceptable that any article in the domain of medical image analysis be published without a full set of source code, data, and parameters that will enable an independent group to replicate the process and verify or refute the findings.

### Conflict of interest statement

The authors declare that the research was conducted in the absence of any commercial or financial relationships that could be construed as a potential conflict of interest.
